# Two Different Heated Tobacco Products vs. Cigarettes: Comparison of Nicotine Delivery and Subjective Effects in Experienced Users

**DOI:** 10.3390/toxics11060525

**Published:** 2023-06-11

**Authors:** Jochen Vukas, Nadja Mallock-Ohnesorg, Tobias Rüther, Elke Pieper, Luna Romano-Brandt, Yvonne Stoll, Lukas Hoehne, Nestor Burgmann, Peter Laux, Andreas Luch, Andrea Rabenstein

**Affiliations:** 1 Department of Psychiatry and Psychotherapy, University Hospital, Ludwig Maximilian University of Munich (LMU), Nussbaumstrasse 7, 80336 Munich, Germanytobias.ruether@med.uni-muenchen.de (T.R.); andrea.rabenstein@med.uni-muenchen.de (A.R.); 2 Department of Chemical and Product Safety, German Federal Institute for Risk Assessment (BfR), Max-Dohrn-Straße 8-10, 10589 Berlin, Germany; elke.pieper@bfr.bund.de (E.P.);

**Keywords:** heated tobacco products, nicotine delivery, craving reduction, IQOS, glo

## Abstract

Heated tobacco products (HTPs) produce aerosol using a different mechanism than tobacco cigarettes, leading to lower emissions of some harmful substances, but also of nicotine as reported by some independent studies. Lower nicotine delivery could lead to compensatory puffing when product use does not sufficiently satisfy cravings. Thus, this three-arm crossover study was conducted to characterize the potential of two different HTPs to deliver nicotine and satisfy cravings compared with conventional cigarettes in users who had already switched to HTPs. Fifteen active, non-exclusive HTP users consumed the study products according to a pre-directed puffing protocol. At predetermined time points, venous blood was sampled and the subjective effects of consumption were assessed. Nicotine delivery by both HTPs was comparable, but significantly lower than that by conventional cigarettes, suggesting a lower addictive potential. Cravings were reduced by all products, with no statistically significant differences between them, despite the different nicotine deliveries. This indicated that HTPs do not necessarily need high nicotine deliveries with high addictive potential, as are characteristic of tobacco cigarettes. These results were followed up on with an ad libitum use study.

## 1. Introduction

**Figure 1:**
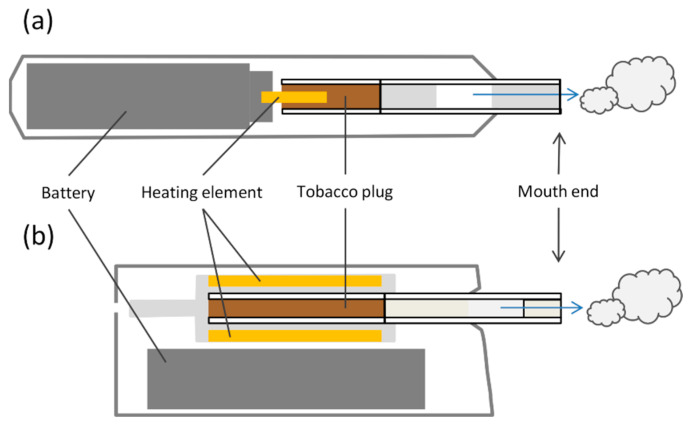
Schematic figures of (**a**) HTP 1 and (**b**) HTP 2 (modified from [2]).

In recent years, various novel tobacco and related products have been introduced to the market that differ from cigarettes in their designs, uses, and emission compositions. One such new product category comprises heated tobacco products (HTP) [1,2]. Although, within this product category, the product design and mechanism to produce aerosol may vary, HTPs have three characteristics in common: (1) they contain (processed) tobacco leaf material; (2) they produce an inhalable, nicotine-containing aerosol; and (3) the tobacco is not directly ignited. The temperature that is applied to the tobacco ranges from room temperature to 350 °C, depending on the device [1,2]. One type of HTP consists of a heating device and separate consumables that resemble cigarettes, which contain a tobacco rod and a filter mouthpiece and are inserted into the device [3,4]. As is schematically shown in Figure 1, the heat might be applied from a source that is inserted into the tobacco plug (Figure 1a, HTP 1) [3] or from the outside (Figure 1b, HTP 2) [4].

HTPs are mostly developed by the tobacco industry and are marketed as harm-reducing alternatives to conventional cigarettes [5]. These claims stems from the differences in emission composition due to the lower temperatures applied to the tobacco. Reduced emission of some carcinogenic compounds by HTPs compared with cigarette smoke was confirmed by independent studies using smoking machines [6,7,8,9,10,11]. Depending on the tobacco cigarette chosen as a reference, some studies have also reported a lower nicotine content in the aerosol [6,7,8,9,11]. However, carcinogens and other toxicologically relevant compounds were still present in the emissions [6,7,8,9,10,11]. In a recent Cochrane review, available data from randomized control trials, all conducted by HTP manufacturers, were evaluated [12]. Authors have concluded that HTPs probably lead to a reduced exposure to toxicants compared with conventional cigarettes, but remarked that exposure may possibly be higher compared with tobacco abstinence [12]. 

When new nicotine delivery products are brought to the market with harm reduction claims, certain aspects need to be determined aside from toxicant exposure. It should be noted that no long-term studies are available to support such claims. Therefore, only potential risk reduction can be discussed. Additionally, a comprehensive potential reduced risk assessment is needed that includes all toxicants present in the emissions, including targeted analyses for substances that are known to come from tobacco products and nontargeted screening approaches for other toxicants that may be present and would not occur in conventional cigarettes. To pose a potential risk-reducing alternative, consumers need to switch completely to the alternative product [13,14]. For this, it is believed that nicotine delivery needs to be sufficiently high to satisfy cravings and to avoid the continuation of simultaneous cigarette use. Dual use has been shown to increase health risks even more than cigarette-only use in the case of dual users of e-cigarettes and cigarettes [15]. Further, increased puffing or increased use to compensate for lower nicotine delivery and craving satisfaction, leading to subsequent increased exposure to harmful substances, has been reported in e-cigarette users [16,17], and most prominently in smokers of “light” cigarettes [18,19]. Thus, nicotine delivery and craving satisfaction, either towards cigarettes or the products to which the consumers have switched, need to be studied. 

On the other hand, high nicotine delivery comes with a downside. These products could induce addiction in naïve users that experiment with them. The addictive potential of a drug is highly correlated with the time between administration and the occurrence of central reward effects in the brain [20,21]. Therefore, it is particularly important to study the nicotine delivery during the acute phase of product use, i.e., the first 5 min [22].

Nicotine delivery of HTPs has mostly been studied by product manufacturers [23,24,25,26], and only a few studies on HTP 1 have been published without conflicts of interest [27,28]. To the best of our knowledge, the two HTPs discussed herein have not yet been compared with each other in a single kinetic study. In most of the mentioned studies, participants were smokers, and were included in order to reflect the transitional phase from cigarettes to HTPs. However, to study whether the products satisfy cravings after this phase, participants should be experienced with HTP use. 

Information on nicotine delivery and craving reduction can be gathered in clinical studies with different designs, regarding, e.g., user experience, study duration, or puffing procedure (pre-directed puffing protocol vs. ad libitum use). The presented study is part of a study series which uses a two-step-approach. At first, the focus was set on nicotine invasion into the blood during the acute phase and craving reduction in already experienced users. The products of concern, i.e., pod e-cigarettes in the first study [29] and HTPs in the presented study, were consumed by experienced users following a pre-directed puffing protocol. The puffing protocol was standardized between studies to allow for comparison of the products and to reduce influences by individual consumers, rather than to reflect actual use. As a second step, the pod e-cigarette, HTP 1, and tobacco cigarettes were investigated in a clinical study with a near real-world ad libitum design, which focused on puffing behavior and nicotine self-titration [30].

In the presented single-center, three-arm crossover study, 15 regular HTP users consumed two different HTPs and a tobacco cigarette on different days, following a pre-directed puffing protocol. The aim of the study was to characterize the product’s potential to deliver nicotine in the acute phase and to relieve product cravings when used by experienced HTP users. Although the study was too small to allow us to draw generalizable public health conclusions, the knowledge gained will help us to perform a science-based risk assessment of the products.

## 2. Materials and Methods

### 2.1. Aims and Ethics

The present study aimed to gather information on the addictive potential and addiction satisfaction of two HTPs compared with a conventional tobacco cigarette. The subjective effects and nicotine delivery into the blood in the acute phase after consumption of these products were assessed using a pre-directed puffing regimen (10 puffs, 3 s puff duration, 30 s puff interval). The study was approved by the ethics committee of the LMU Munich (No. 21-0231), and was performed in accordance with the principles of the Declaration of Helsinki in the currently valid version. The study was registered at the DRKS (DRKS00024596). Informed consent was obtained from all participants prior to participation in the study.

### 2.2. Study Products and Groups

The study was designed as a single-center, three-arm crossover study. The products studied were (a) a commercial, combustible tobacco cigarette (Marlboro Red, Philip Morris); (b) HTP 1 (IQOS 3 DUO with “Amber Heets” sticks); and (c) HTP 2 (glo with “neo Tobacco Bright” sticks). Participants used their own devices if available; otherwise, the device was provided. HTP 1 was purchased at a local licensed store in Munich; HTP 2 was purchased from the officially licensed online shop. 

### 2.3. Participants

Participants were recruited during April and May 2021 via Facebook, the intranet of the university, word of mouth, and mailings. All subjects were enrolled in the study after inclusion criteria and exclusion criteria had been checked and participants had provided written informed consent. Fifteen subjects (dual users, seven male, eight female) fulfilled all requirements for study participation and completed all three sessions with the study products. All sessions were separated by a minimum of 48 h. This resulted in a total of 45 experimental sessions. A power analysis for the QSU with 2 measurements was calculated with the following parameters: effect size f, 0.25; correlation between repeated measures, 0.5; nonsphericity correction ε, 1. As the required total sample size was 42, 15 participants were recruited for the three-arm study, resulting in a total sample size of 45. 

The inclusion criteria for all volunteers were as follows: aged between 18 and 55 years; 12 h of abstinence (nicotine and tobacco cigarette consumption); CO levels < 5 ppm (measurement in the expiratory air using a micro-smokerlyzer (Bedfont Scientific Ltd., Anif, Austria) to verify smoking abstinence; nicotine plasma levels at baseline ≤ 10 ng/mL to verify abstinence from other nicotine products; and the ability to give consent. The special inclusion criteria were HTP use for at least 3 months with daily consumption and occasional consumption of conventional tobacco cigarettes for a minimum of 3 months, with a maximum of 20 cigarettes per week.

The exclusion criteria were as follows: under 18 or over 55 years of age; acute psychiatric illness according to ICD-10/DSM IV; other serious psychiatric disorders; acute suicidality; existing pregnancy; breastfeeding; drug, medication, or alcohol abuse at the time of the study; malignant cancer in the past 5 years; serious internal illness, especially cardiovascular diseases such as manifest arterial hypertension; severe heart disease (DCM, history of heart attack); pacemaker implantation; respiratory failure; severe active infectious disease; and current tobacco or smoking cessation therapy.

### 2.4. Study Design and Questionnaires

Clinical data were collected during April and May 2021. Four appointments were scheduled which the test subjects could attend in order to participate in the study. The first appointment was considered a screening; the actual study sessions and measurements took place at the second, third, and fourth appointments. Participants’ usual smoking and HTP use behavior was evaluated with standardized and modified questionnaires, respectively. 

With the initial questionnaire at the screening appointment, sociodemographic data such as sex, age, weight, and known pre-existing illnesses, as well as smoking and HTP use behavior, were assessed. Furthermore, physical dependence was assessed for all 3 tested products using the Fagerström Test of Nicotine Dependence (FTND) according to Heatherton et al. [31]. As no validated version of the FTND exists for HTPs, an adapted, but non-validated, questionnaire was used for these products (see Supplementary Materials). 

**Figure 2:**
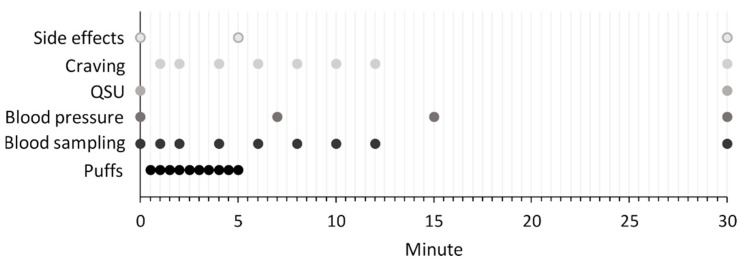
Study design illustrating measurements and their time points.

The consumption sessions were carried out as described in Figure 2. A puffing regimen with a puff duration of 3 s and interpuff intervals of 30 s was selected according to a previous study on e-cigarettes [29]. The puffing regime was chosen to allow for comparison between studies rather than to represent the usage pattern of an average HTP user. All HTP devices were fully charged prior to use. In total, ten puffs were taken. A metronome was used to standardize the duration of the inhalations by providing an acoustic signal at the beginning and at the end of each inhalation. The study investigator instructed all study participants to inhale in exactly the same way at each inhalation and study visit. Participants were instructed to inhale the product aerosols into their lungs. Blood was sampled for a total of nine blood samples. The participants’ heart rates and blood pressure were measured at four time points: at baseline, after 8 min, after 15 min, and after 30 min. 

The German version of the Questionnaire on Smoking Urges (QSU-G), by Müller et al., was used to assess cravings both before and immediately after each session [32]. As no validated version of a test on smoking urges exists for HTPs, an adapted and non-validated version for these products was used. The questionnaire was modified by rephrasing the items to denote the use of the respective HTP instead of cigarettes (see Supplementary Materials). 

At baseline, after 5 min, and after 30 min, participants rated side effects on a visual analog scale (VAS) ranging from 0 (no effect) to 10 (strongest effect). The questionnaire included the side effects of drowsiness, mouth irritation, throat irritation, dizziness, salivation, cold hands/feet, cardiac palpitation, headache, sweating, nausea, and feeling of vomiting.

### 2.5. Blood Sampling and Determination of Nicotine, Cotinine, and Hydroxycotinine Plasma Levels

A total of 9 blood samples of 7.5 mL each were drawn at the time points illustrated in Figure 2 using peripheral venous Safety Multifly cannulas and S-Monovettes. Blood sampling was performed in accordance with the generally applicable hygienic standards. Blood samples were cooled until centrifugation (1500× *g*, 10 min, 4 °C). To 990 µL plasma, 10 µL internal standard mix (500 ng/mL nicotine-d_3_, cotinine-d_3_, hydroxycotinine-d_3_ in acetonitrile) at LMU in Munich, which occurred prior to storage at −80 °C and shipment to BfR in Berlin. Plasma levels of nicotine, cotinine, and trans-3′-hydroxycotinine (hydroxycotinine) were determined at BfR with a validated, previously published method using liquid chromatography–tandem mass spectrometry (LC-MS/MS) following protein precipitation [33]. A matrix-matched calibration was used for quantification. 

### 2.6. Pharmacokinetic (PK) Parameters and Statistical Analysis

Prior to the calculation of PK parameters, baseline nicotine levels (nicotine levels directly before product consumption) were subtracted. Areas under the plasma concentration–time curve (AUC) were calculated by applying the linear trapezoid rule. For C_max_, the highest achieved plasma nicotine level per curve was used; for t_max_, the according time point was used. The geometric mean, coefficient of variance (CV), and Bonferroni-corrected two-sided paired *t*-test with lognormal values were used for the statistical analysis of AUC and C_max_. For the mean plasma curves, arithmetic means and 95% confidence intervals (CI) were calculated. For t_max_, the median and range were calculated, and statistical significance was tested with a two-sided paired *t*-test. To calculate the nicotine metabolite ratio (NMR), which was used as a surrogate for CYP 2A6 activity, the hydroxycotinine plasma level was divided by the cotinine plasma level (baseline levels at the first visit) [34,35]. A cut-off value of 0.31 was used to distinguish between slow (NMR < 0.31) and normal or rapid (NMR > 0.31) nicotine metabolizers, as was established by Lerman et al. [36] for their clinical study. For the participant characteristics, median and interquartile ratios (IQR) were calculated. The statistical significance of the QSU-G scores was tested with a two-sided paired *t*-test. For all statistical analyses for which no sphericity could be assumed, a Greenhouse–Geisser correction was applied. Statistical analysis was performed with the Statistical Package for Social Sciences (SPSS), version 26.0.

## 3. Results

### 3.1. Participants

The characteristics of the 15 participants in the study are summarized in Table 1, including information on age, sex, FTND score, NMR, and cigarette/HTP use behavior. The FTND scores varied from very low to very severe physical nicotine dependence. Ten participants had a very low level, four had a moderate level, and one participant had a severe level of dependence. By using an NMR cut-off value of 0.31 to distinguish between normal/rapid (NMR > 0.31) and slow (NMR < 0.31) nicotine metabolizers [36], three out of fifteen participants were classified as slow metabolizers.

### 3.2. Nicotine Delivery

**Figure 3:**
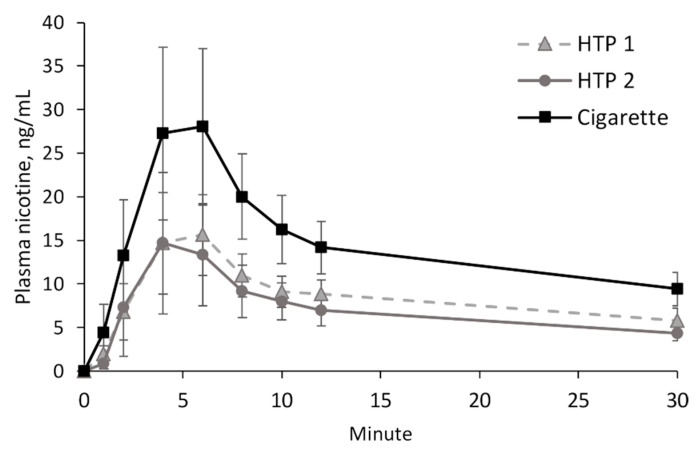
Arithmetic means and 95 % confidence interval of nicotine plasma curves after consumption of the study products.

Mean plasma nicotine curves after consumption of the study products are displayed in Figure 3, and the PK parameters C_max_, AUC, and t_max_, including a statistical evaluation, are shown in Table 2. The nicotine delivery after consumption of both HTPs was not statistically different, with slightly lower AUC and C_max_ after use of HTP 2. The consumption of a cigarette led to a statistically significantly higher level of nicotine delivery compared with both HTPs (*p* < 0.005). Compared with HTP 2, AUC and C_max_ were more than twice as high after cigarette consumption. Individual plasma nicotine concentrations are presented in Tables S1–S3 and are summarized in Figure S1 in the Supplementary Materials.

### 3.3. Nicotine Delivery in the Acute Phase

**Figure 4:**
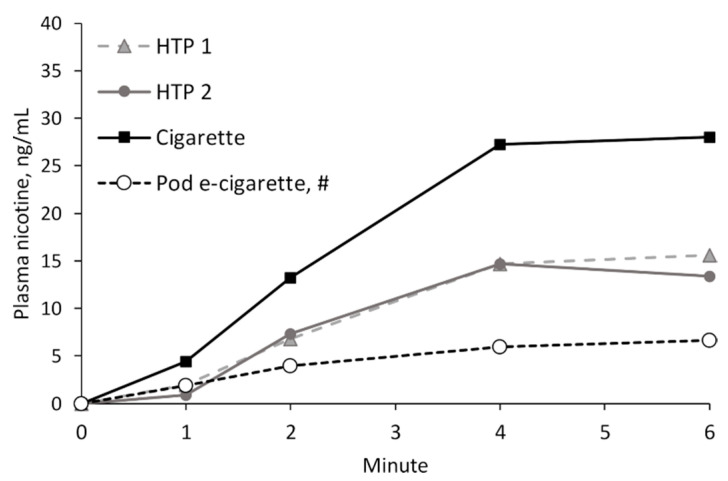
Arithmetic means of nicotine plasma curves during the acute phase after consumption of the study products, in comparison with previously published results from [29] for a pod e-cigarette.

A magnification of the mean plasma nicotine curves in the first minutes of product consumption (acute phase) is displayed in Figure 4. While the differences between the two HTPs were minor, cigarette consumption led to a much steeper increase in plasma nicotine levels. All three plasma nicotine curves flattened after 4 min.

In a previous study on nicotine kinetics, which considered the acute phase after consumption of both the new and an older version of a pod e-cigarette with 18 mg/mL nicotine, participants (all experienced e-cigarette users) were instructed to use the same pre-directed puffing regime as that used in the present study [29]. The results for the new version of the pod e-cigarette are included in Figure 4 in order to compare the increases in plasma nicotine concentrations in the two studies. Consumption of the pod e-cigarette led to the slowest increase in blood nicotine levels in the acute phase.

### 3.4. Relief of Cravings and Urges to Use the Product

**Figure 5:**
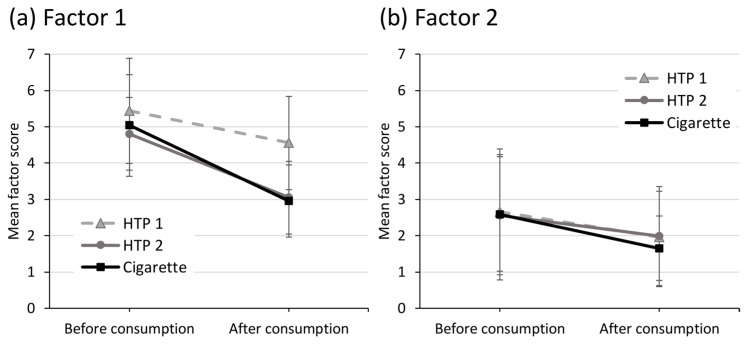
Mean scores for (**a**) positive reinforcement and (**b**) negative reinforcement of urges to use the respective study product before and after consumption (mean and standard deviation).

Changes in smoking urges before and after consumption (at 30 min) were measured using the QSU-G for cigarettes and a modified version, which assessed the urges to use the respective HTP. The results are displayed in Figure 5. Factor 1 summarizes positive reinforcement factors, such as the intention to use the product or the anticipation of positive effects. The mean score of factor 1 decreased significantly (all *p* < 0.05) after using all study products. The differences in the levels of reduction between the three products were only trends, and were not statistically significant (*p* = 0.119). Factor 2 summarizes negative reinforcement factors, such as cravings to use the product or the anticipation of relief from withdrawal symptoms. The mean score of factor 2 also decreased significantly (all *p* < 0.05) in all groups. Again, the differences between products were not statistically significant.

**Figure 6:**
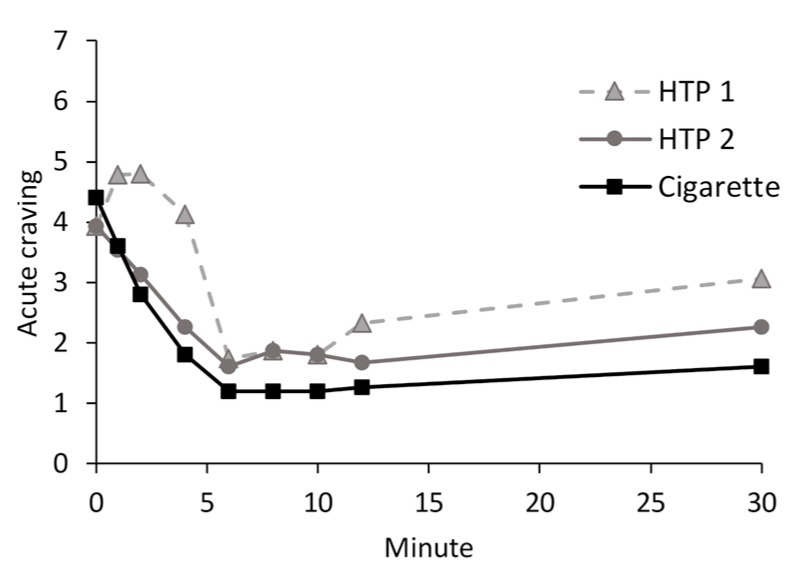
Acute cravings for the tested product (single question answered on a scale from 1 to 7) over the study period.

While the complete questionnaire was used only at the baseline and at the end of the study period, an item concerning acute cravings for the respective product was asked at each time point of blood sampling. The results are shown in Figure 6. Acute cravings to use HTP 2 or cigarettes decreased during consumption, and was at its lowest immediately after product use. Acute cravings for HTP 1 were also at their lowest immediately after product consumption. As the study progressed, acute cravings increased in all groups. The reductions in acute cravings did not differ significantly between the products. Individual scores for acute cravings are given in Tables S4-S6 in the Supplementary Materials.

### 3.5. Side Effects

**Figure 7:**
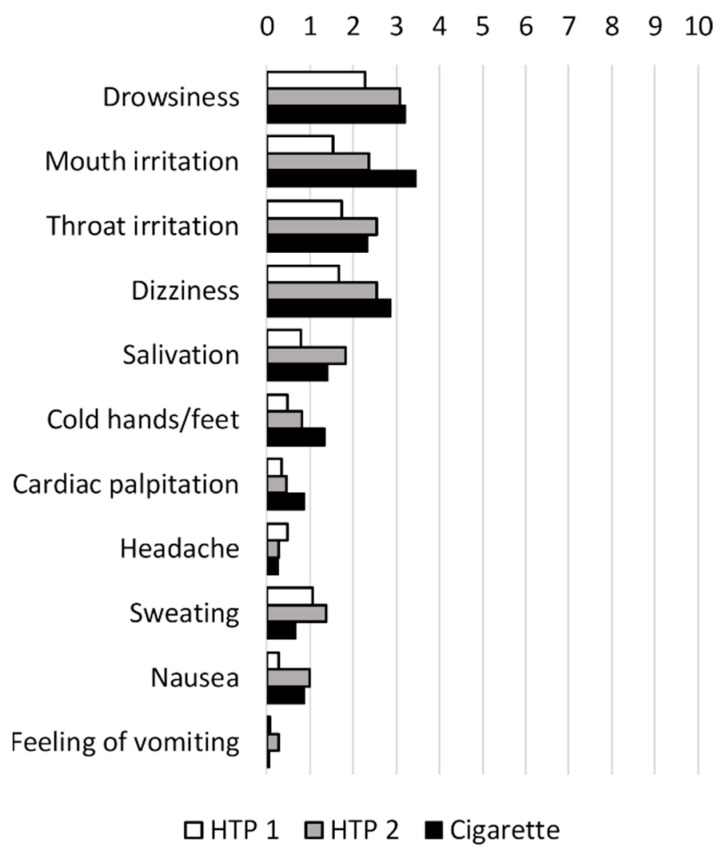
Side effects reported at 5 min on a visual analog scale (VAS), ranging from 0 (no effect) to 10 (strong effect).

Participants were asked to rate the side effects which they experienced on a VAS from 0 (no effect) to 10 (strong effect). The side effects reported directly after product use are shown in Figure 7. Overall, side effects were low. The side effects with the highest scores in all groups were drowsiness, mouth irritation, throat irritation, and dizziness. Other reported side effects were dry mouth, tiredness, tussive irritation, feeling of mucus-ridden lung, dragging pain behind the sternum, and shaking hands. Ratings of side effects inquired at baseline and after 30 min are presented in Figure S2 in the Supplementary Materials.

## 4. Discussion

The presented study was conducted with two aims. Firstly, nicotine delivery with a focus on nicotine invasion in the acute phase of consumption was studied for two HTPs in comparison with tobacco cigarettes to gain insight into the addictive potential of these products. The results were compared with those of a previous study on a pod-type e-cigarette, in which the same pre-directed puffing protocol was used. Secondly, subjective effects, especially reductions in product cravings, were assessed in order to shed light on the level of satisfaction achieved by experienced HTP users. 

The highest nicotine concentration and uptake were achieved after consumption of tobacco cigarettes, with a C_max_ of 25.1 ng/mL and an AUC_0–30 min_ of 6.1 ng/mL×h, as expected. Nicotine delivery by both HTPs did not show any significant differences, although there were differences in their heating mechanisms (see Figure 1). After consumption of HTP 1, a C_max_ of 14.9 ng/mL and an AUC_0–30 min_ of 4.0 ng/mL × h were achieved. After consumption of HTP 2, these values were 11.6 ng/mL and 3.0 ng/mL × h. It should be noted that the present study used a pre-defined puffing protocol that did not allow for individualized product use. Individually chosen consumption patterns may have led to different nicotine kinetics.

The manufacturers of HTP 1 and HTP 2 have published nicotine kinetics studies comparing their products with cigarettes [23,24,25]. In one study on HTP 1, the nicotine deliveries after a single use of a cigarette (C_max_ 11.9 ng/mL) or HTP 1 (C_max_ 8.4 ng/mL) were lower compared with the results presented herein [23]. The level of craving reduction was the same for both products [23]. In another study on HTP 1, the cigarettes and the HTPs yielded comparable C_max_ values, with 13.82 ng/mL and 14.30 ng/mL for the cigarettes and HTP 1, respectively [24]. The craving reductions were also comparable between the products. While the nicotine delivery of HTP 1 was comparable to the results presented herein, the nicotine delivery of the cigarette was much lower [24]. The participant numbers were 28 [23] and 60 [24]. The studies were conducted in Ireland (with Caucasian participants, mean FTND score: 4.9) [23] and in Japan (no FTND reported) [24], using cigarette smokers with little HTP use experience. In a study on HTP 2, the C_max_ values were similar to the findings presented herein, with 22.7 ng/mL for the cigarette and 8.6 ng/mL and 10.5 ng/mL for HTP 2, which was found using two different heat sticks [25]. The study was conducted in Italy using 32 cigarette smokers with little HTP use experience (mean FTND score: 6.0) [25]. In all three studies, the puffing regimens were not pre-directed [23,24,25].

In other studies, HTP 1 was compared with cigarettes and the same pod e-cigarette as mentioned above. Phillips-Waller et al. allowed 22 daily e-cigarette users (without HTP experience; on average smoking < 1 cigarette/day) to consume the products for 5 min ad libitum [27]. The pod e-cigarette, i.e., the US version with 59 mg/mL nicotine, showed a similar level of nicotine delivery compared with tobacco cigarettes. HTP 1 had the lowest C_max_, with 8.3 ng/mL [27]. Maloney et al. compared HTP 1 and the US pod e-cigarette with 59 mg/mL nicotine in two sets, i.e., controlled (10 puffs, ~30 s interval) and ad libitum (over 90 min), in 18 cigarette smokers with no experience with pod e-cigarettes or HTPs (eight Caucasian, seven African-American, three other) [28]. They came to similar conclusions as the present study when using the controlled puffing parameters, with C_max_ values of 20.4 ng/mL and 12.7 ng/mL for cigarettes and HTP 1, respectively. Cigarettes and HTP 1 both reduced cravings [28]. The manufacturer of the pod e-cigarette has tested different versions of their e-cigarette in comparison with HTP 1 and cigarettes in smokers (half of them being ever-users of electronic nicotine delivery systems) in two sets: controlled (10 puffs, 30 s intervals, 3 s puff duration, 32 participants) and ad libitum (over 10 min, 30 participants) [26]. The controlled use yielded similar results to the ones presented herein, with C_max_ values of 24.83 ng/mL and 13.68 ng/mL for cigarettes and HTP 1, respectively. The pod e-cigarette version with 18 mg/mL nicotine had a lower level of nicotine delivery compared with cigarettes and HTP 1, both overall and in the acute phase [26].

None of these studies have directly compared nicotine delivery from the two HTPs. However, nicotine yields have been compared using a smoking machine. A study by the manufacturer of HTP 2 yielded 12 puffs with a total of 1.16 mg nicotine from HTP 1 and 8 puffs with a total of 0.462 mg nicotine from HTP 2 by applying a modified Health Canada Intense puffing regime (55 mL puff volume, 2 s puff duration, 30 s interval, no blockages of ventilation holes) [37]. In another study by independent researchers using the Health Canada Intense (HCI) and ISO 3308 puffing regime (35 mL puff volume, 2 s puff duration, 60 s interval), HTP 1 yielded 0.1 mg nicotine/puff and 0.07 mg nicotine/puff with the HCI and the ISO 3308 regimes, respectively [11]. For HTP 2, the nicotine yields were lower, with 0.05 mg/puff and 0.03 mg/puff using the HCI and the ISO 3308 regimes, respectively [11]. Based on these results, one would have expected that the HTPs would show different nicotine delivery levels in the clinical setting, especially when the same instructed puffing protocol was used for both. However, the findings presented herein are in contrast to this. While in this clinical study, the number, duration, and intervals of puffs were pre-directed, the puff volume was not controlled. It is possible that participants used different puff volumes across the study arms, potentially influencing the results.

The steepness of the nicotine curves in the acute phase of consumption was compared between products to draw conclusions on the addictive potential of these products. It is believed that one reason why cigarettes are such addictive products lies in their rapid delivery of nicotine to the blood and subsequently to the brain. Thus, if other nicotine delivery products showed similar kinetics, it could indicate a similar level of addictiveness of the products, although one should be aware that other factors play important roles as well. Nicotine addiction should be considered as a complex phenomenon, and psychosocial and biogenic factors, for example, should also be taken into account [38]. In the long term, increasing sensitivity and responsiveness to smoking stimuli (approach tendency) will also play a role in the development of tobacco dependence [39,40]. Both HTPs led to a slower invasion of nicotine into the blood compared with tobacco cigarettes. From a pod e-cigarette (18 mg/mL nicotine) that was investigated in a previous study with different participants, but which used the same puffing protocol, nicotine delivery in the acute phase was slower than by both HTPs. This indicates that the addictive potential might increase in the following order: the studied pod e-cigarette < both studied HTPs < tobacco cigarettes. It should be noted that the product categories of e-cigarettes and HTPs themselves are very heterogeneous. For example, HTPs have different heating mechanisms, and the compositions of the tobacco sticks can widely differ. Additionally, knock-off products are available. Thus, the addictive potential of other e-cigarettes or HTPs could be different. Further, it should be kept in mind that the potentially lower addictiveness of HTPs compared with cigarettes does not indicate that the products are not addictive at all, or are harmless. 

Regardless of nicotine kinetics, a reduction in cravings to use the product again was reported after the use of all studied products. Although the reduction in factors of positive reinforcement, e.g., the expectation to obtain a pleasant sensation from product use, was less pronounced after the use of HTP 1, the differences between products were not statistically significant. With a larger sample size, it is conceivable that the trend difference between HTP 1 and the other two products might have become statistically significant. This is a relevant point to be addressed in future studies. As a limitation of this assessment, if participants liked a product less, this could affect the baseline values of QSU. The selected participants were all experienced HTP users, which was intended to reduce this effect; this is also reflected in the overall low side effects. The study was designed to test the satisfaction of regular HTP users, and not to reflect the transition from cigarettes to HTPs. Further, it should be noted that the questionnaire utilized to assess smoking urges was designed and validated for cigarettes. The adapted versions inquiring about reinforcement factors for use of the HTPs were not validated. 

However, the fact that product cravings were reduced despite the lower nicotine delivery indicates that it might not be necessary for HTPs to have the same high nicotine delivery as tobacco cigarettes. This means that alternative nicotine delivery products would not need to, and, consequently, should not, possess the same nicotine kinetics as cigarettes. This would help to reduce the addictive potential of new products. Further investigation on this is necessary. The dual use of HTPs and tobacco cigarettes is problematic, and adequate regulatory measures could reduce incentives to use HTPs where cigarettes cannot be used. 

New products should aim toward achieving the lowest addictive potential possible. One argument justifying the high nicotine concentrations of alternative products is that compensatory puffing or increased product use should be avoided [16,17]. For HTPs, this would mean that the lower nicotine delivery could result in a higher consumption of heat sticks compared with cigarettes. Increased consumption of heat sticks would be accompanied with increased exposure to the harmful substances that are present in the aerosol. Therefore, the next step was to study the use of HTPs and cigarettes ad libitum over 90 min. The number of cigarettes smoked and heat sticks consumed were compared in order to follow up on the results presented herein [30].

## Figures and Tables

**Table 1:** Participant characteristics, including cigarette and heated tobacco product (HTP) use behavior.

Participant Characteristics	
Age, median (IQR)	32 (20)
Sex, female, n (%)	8 (53)
Sex, male, n (%)	7 (47)
Fagerstrom Test for Nicotine Dependence (FTND) *, median (IQR)	2 (3)
Nicotine metabolite ratio (NMR), median (IQR)	0.46 (0.28)
Number of days on which cigarettes were smoked within the last 30 days, median (IQR)	4.5 (9)
Number of cigarettes smoked per day when cigarettes were smoked, median (IQR)	3 (2.5)
Number of days on which the HTP was used within the last 30 days, median (IQR)	30 (0)
HTP sticks used per day, median (IQR)	11 (5)

* FTND, modified for HTPs.

**Table 2:** Summary of relevant PK parameters and statistical evaluation for the two heated tobacco products (HTP) and tobacco cigarettes.

	HTP 1	HTP 2	Cigarette	HTP 1 vs. HTP 2	HTP 1 vs. Cigarette	HTP 2 vs. Cigarette
C_max_ (ng/mL)	14.9 (70%)	11.6 (137%)	25.1 (87%)	*p* = 0.540	*p* = 0.021 *	*p* = 0.010 *
AUC_0–30 min_ (ng/mL × h)	4.0 (46%)	3.0 (84%)	6.1 (64%)	*p* = 0.279	*p* = 0.006 *	*p* = 0.001 **
t_max_ (min)	6 (4–10)	6 (4–12)	6 (4–6)	*p* = 1.114	*p* = 0.288	*p* = 0.081

C_max_ and AUC: geometric mean and coefficient of variance (CV%), *p*-values obtained with Bonferroni-corrected, paired, two-sided *t*-test with lognormal values. t_max_: median and range, *p*-values obtained with Bonferroni-corrected pairing, two-sided *t*-test. * and **: statistically significant (* *p* < 0.05, ** *p* < 0.005).

## Data Availability

The data presented in this study are available in the Supplementary Materials.

## Supplementary Materials

The following supporting information can be downloaded at: https://www.mdpi.com/article/10.3390/toxics11060525/s1, Figure S1: Individual plasma nicotine curves after consumption of (a) HTP 1, (b) HTP 2, or (c) a conventional cigarette with baseline correction; Figure S2: Side effects reported at (a) baseline, (b) 5 min, or (c) 30 min on a visual analog scale (VAS) ranging from 0 (no effect) to 10 (strong effect). Tables S1–S3: Nicotine plasma concentrations of individual participants before baseline correction after use of HTP 1, HTP 2, or cigarettes with their nicotine metabolic ratios (NMR); Tables S4–S6: Acute craving for HTP 1, HTP 2, or cigarettes (single question answered on a scale from 1 to 7) over the study period per participant.
