# Supplementary material for: Two Different Heated Tobacco Products vs. Cigarettes: Comparison of Nicotine Delivery and Subjective Effects in Experienced Users
Source: Toxics. 2023 Jun 11;11(6):525. doi: 10.3390/toxics11060525 (PMC10301154; doi:10.3390/toxics11060525)
Supplement: Supplementary file 1 [file toxics-11-00525-s001.zip › toxics-2327783-supplementary.pdf]

Supplementary Material

Two Different Heated Tobacco Products vs. Cigarettes: Comparison of Nicotine Delivery and Subjective Effects in Experienced Users

Jochen Vukas [1,†], Nadja Mallock-Ohnesorg [2,*,†], Tobias Rüther [1], Elke Pieper [2], Luna Romano-Brandt [1], Yvonne Stoll [1], Lukas Hoehne [1], Nestor Burgmann [1], Peter Laux [2], Andreas Luch [2] and Andrea Rabenstein [1]

[1] Department of Psychiatry and Psychotherapy, University Hospital, Ludwig Maximilian University of Munich (LMU), Nussbaumstrasse 7, 80336 Munich, Germany; tobias.ruether@med.uni-muenchen.de (T.R.); andrea.rabenstein@med.uni-muenchen.de (A.R.)
[2] German Federal Institute for Risk Assessment (BfR), Department of Chemical and Product Safety, German Federal Institute for Risk Assessment (BfR), Max-Dohrn-Straße 8-10, 10589 Berlin, Germany; elke.pieper@bfr.bund.de (E.P.)
* Correspondence: nadja.mallock@bfr.bund.de
† These authors contributed equally to this work.

Citation: Vukas, J.; Mallock-Ohnesorg, N.; Rüther, T.; Pieper, E.; Romano-Brandt, L.; Stoll, Y.; Hoehne, L.; Burgmann, N.; Laux, P.; Luch, A.; et al. Two Different Heated Tobacco Products vs. Cigarettes: Comparison of Nicotine Delivery and Subjective Effects in Experienced Users. *Toxics* **2023**, *11*, 525. https://doi.org/10.3390/toxics11060525

Academic Editor: Kenneth M. Taylor

Received: 21 March 2023
Revised: 29 May 2023
Accepted: 9 June 2023
Published: 11 June 2023

Copyright: © 2023 by the authors. Submitted for possible open access publication under the terms and conditions of the Creative Commons Attribution (CC BY) license (https://creativecommons.org/licenses/by/4.0/).

Table of contents

1. .... Modified questionnaires 2
2. .... Nicotine plasma concentrations of individual participants 4
3. .... Craving 6
4. .... Side effects 8

1. Modified questionnaires

Modified versions of Fagerstrom Test of Nicotine Dependence (FTND) and Questionnaire on Smoking Urges (QSU-G) in German were used to fit the research question as presented below. Rephrasing of items was kept at a minimum, scoring and interpretation remained the same.

Modified FTND:

1. Wann nach dem Aufwachen konsumieren Sie Ihren ersten Stick?
   - Innerhalb von 5 Minuten (3)
   - Nach 6-30 Minuten (2)
   - Nach 31-60 Minuten (1)
   - Nach 60 Minuten (0)
2. Finden Sie es schwierig, auf die IQOS/ Glo zu verzichten, wenn es verboten ist? (z. B. im Kino, in Versammlungen usw.)
   - Ja (1)
   - Nein (0)
3. Auf welchen Stick würden Sie nicht verzichten wollen?
   - Den 1. Stick morgens (1)
   - Jeden anderen Stick (0)
4. Wie viele Sticks konsumieren Sie am Tag?
   - 0-10 (0)
   - 11-20 (1)
   - 21-30 (2)
   - 31 und mehr (3)
5. „Rauchen" bzw. konsumieren Sie am Morgen im Allgemeinen mehr als am Rest des Tages?
   - Ja (1)
   - Nein (0)
6. Kommt es vor, dass Sie IQOS/ Glo nutzen, wenn Sie krank sind und tagsüber im Bett bleiben müssen?
   - Ja (1)
   - Nein (0)

Modified QSU-G (either IQOS or glo, depending on the product used that experimental day):

1. Eine IQOS/ Glo zu nutzen würde mir jetzt dazu verhelfen, mich sehr gut zu fühlen.
2. Ich wäre weniger reizbar, wenn ich jetzt eine IQOS/ Glo nutzen könnte.
3. Nichts wäre besser, als jetzt eine IQOS/ Glo zu nutzen.
4. Mir fehlt die IQOS/ Glo jetzt gerade ***nicht***.
5. Ich werde eine IQOS/ Glo nutzen, sobald ich wieder die Möglichkeit dazu bekomme.
6. Ich möchte jetzt ***nicht*** IQOS/ Glo nutzen.
7. Eine IQOS/ Glo würde meine schlechte Stimmung deutlich verbessern.
8. Eine IQOS/ Glo würde mir jetzt ***nicht*** helfen, mich zu beruhigen.
9. Wenn ich jetzt eine IQOS/ Glo angeboten bekomme, würde ich sie sofort verwenden.
10. Ich könnte ab sofort für eine lange Zeit auf das Verwenden von IQOS/ Glo verzichten.
11. Jetzt eine IQOS/ Glo zu nutzen, wäre ***nicht*** angenehm.

12. Wenn ich jetzt im Moment eine IQOS/ Glo würde nutzen, wäre ich weniger gelangweilt.
13. Das Einzige, was ich jetzt gerne hätte, wäre eine IQOS/ Glo.
14. Wenn ich jetzt eine IQOS/ Glo nutzen würde, würde ich mich weniger müde fühlen.
15. Jetzt einen Stick zu konsumieren würde mich glücklicher machen.
16. Sogar, wenn es jetzt möglich wäre, würde ich IQOS / Glo wahrscheinlich ***nicht*** verwenden.
17. Ich habe jetzt gerade keinen Wunsch nach IQOS/ Glo.
18. Mein Wunsch nach einer IQOS/ Glo scheint gerade so stark zu sein, dass er mich zu überwältigen droht.
19. Wenn ich jetzt eine IQOS/ Glo nutzen würde, erschiene mir fast alles in Ordnung.
20. Ich sehne mich gerade nach einer IQOS/ Glo.
21. Ich würde eine IQOS/ Glo jetzt ***nicht*** genießen.
22. Eine IQOS/ Glo würde jetzt ***nicht*** gut schmecken.
23. Ich verspüre jetzt gerade den Drang nach einer IQOS/ Glo.
24. Ich hätte die Dinge gerade besser im Griff, wenn ich eine IQOS/ Glo nutzen würde.
25. Ich werde eine IQOS/ Glo nutzen, sobald ich die Möglichkeit dazu habe.
26. Ich würde mich körperlich ***nicht*** besser fühlen, wenn ich jetzt einen Stick konsumieren würde.
27. Eine IQOS/ Glo wäre jetzt ***nicht*** sehr befriedigend.
28. Wenn ich jetzt eine IQOS/ Glo in der Hand hätte, würde ich sie wahrscheinlich gar ***nicht*** nutzen.
29. Wenn ich jetzt einen Stick konsumieren würde, könnte ich klarer denken.
30. Ich würde fast alles tun, um jetzt eine IQOS/ Glo zu bekommen.
31. Ich muss jetzt einen Stick konsumieren.
32. Gerade jetzt habe ich ***nicht*** vor eine IQOS/ Glo zu nutzen.

2. Nicotine plasma concentrations of individual participants

Individual nicotine plasma curves after baseline correction are displayed in Figure S1. Nicotine plasma concentrations at the different time points are listed in Tables S1 (for HTP 1), S2 (for HTP 2), and S3 (for conventional cigarette).

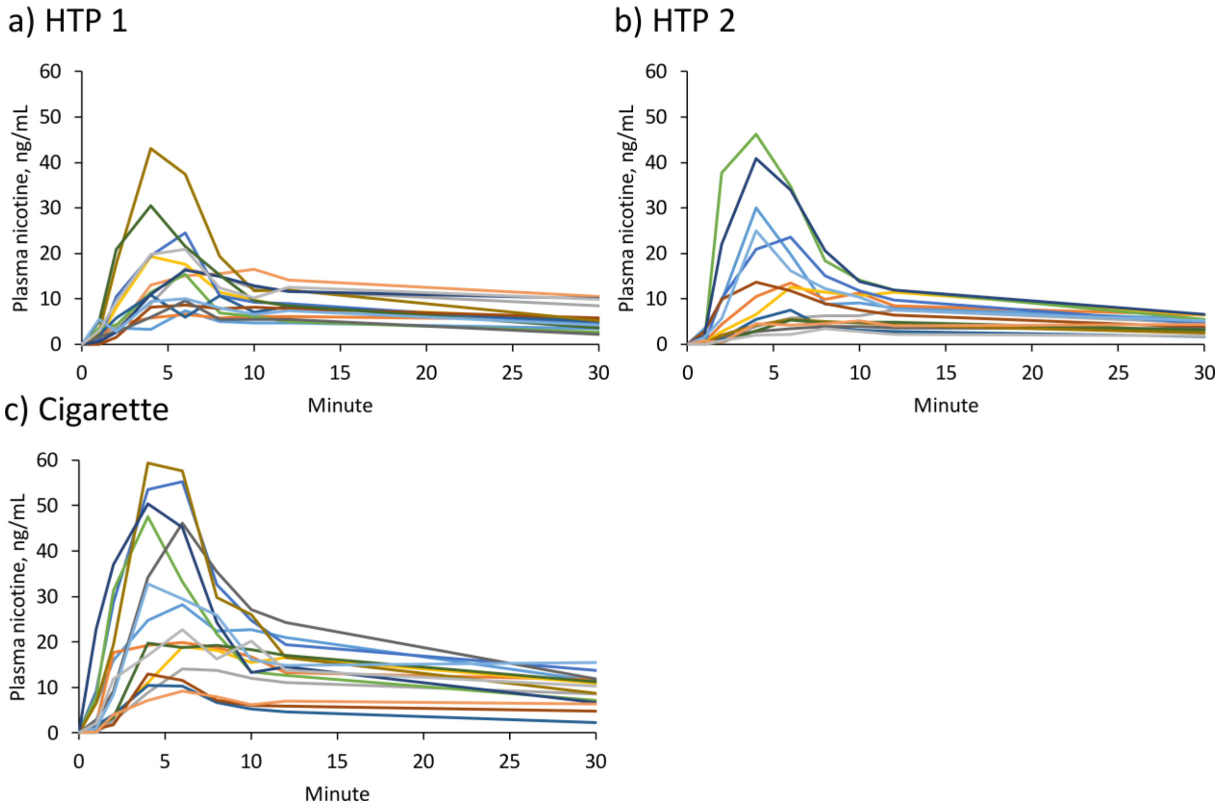

Figure S1. Individual plasma nicotine curves after consumption of a) HTP 1, b) HTP 2, or c) a conventional cigarette with baseline correction.

Table S1. Nicotine plasma concentrations of individual participants before baseline correction after use of HTP 1 with their nicotine metabolic ratios (NMR).

Participant	NMR	0 min	1 min	2 min	4 min	6 min	8 min	10 min	12 min	30 min
1	0.43	0.5	0.7	3.5	13.5	15.6	16.0	17.1	14.6	11.1
2	0.17	1.6	2.8	5.3	4.9	9.1	6.5	6.4	6.2	5.0
3	0.16	4.9	6.7	9.3	10.8	11.5	10.7	11.0	11.1	10.2
4	0.44	1.6	2.1	5.6	10.5	18.3	16.5	13.9	13.3	10.1
5	0.61	0.8	2.1	9.0	20.2	18.5	12.3	10.6	9.2	6.2
6	0.67	0.5	1.5	11.1	20.0	25.1	11.3	9.8	9.5	4.9
7	0.52	0.1	3.6	4.3	11.6	15.5	7.0	6.2	5.2	2.8
8	0.67	0.1	2.1	5.9	10.9	6.1	10.8	7.1	8.5	5.1
9	0.26	0.3	0.2	1.9	8.5	9.0	8.2	8.6	8.2	6.2
10	0.70	0.7	2.4	3.4	6.7	10.3	6.1	6.1	6.1	3.0
11	0.33	0.0	2.7	17.8	43.1	37.5	19.3	11.9	12.0	5.2
12	0.46	0.4	0.9	3.1	11.3	16.8	15.3	13.2	12.0	10.4
13	0.51	2.4	7.4	23.4	32.9	24.0	17.8	12.0	10.9	6.1
14	0.61	1.5	7.0	4.3	10.9	11.7	9.5	8.1	9.0	5.8
15	0.37	0.0	2.9	9.5	19.8	20.8	12.4	10.2	12.6	9.9

Table S2. Nicotine plasma concentrations of individual participants before baseline correction after use of HTP 2 with their nicotine metabolic ratios (NMR).

Participant	NMR	0 min	1 min	2 min	4 min	6 min	8 min	10 min	12 min	30 min
1	0.43	2.8	6.2	12.4	32.9	22.5	11.6	11.9	10.9	8.4
2	0.17	2.2	2.8	6.6	12.8	15.7	12.2	13.5	10.7	8.6
3	0.16	2.2	2.3	3.0	6.1	8.0	8.5	8.5	9.7	6.9
4	0.44	0.4	0.6	3.3	7.0	13.0	11.9	11.1	11.8	6.8
5	0.61	1.2	1.6	11.3	22.1	24.7	16.3	13.0	11.0	5.8
6	0.67	0.1	0.6	37.8	46.3	34.7	18.4	14.3	11.9	5.5
7	0.52	0.1	0.4	1.3	5.7	7.7	3.8	3.3	2.9	1.9
8	0.67	0.0	2.8	9.9	13.7	11.8	8.9	7.6	6.5	3.8
9	0.26	0.4	0.5	2.1	3.2	3.8	4.3	4.4	4.0	3.8
10	0.70	0.7	1.1	2.8	5.0	6.2	5.8	5.4	4.9	3.2
11	0.33	0.0	1.9	22.1	40.9	34.0	20.7	13.9	12.0	6.6
12	0.46	0.7	0.7	1.2	3.7	5.9	5.3	5.5	5.5	3.8
13	0.51	2.8	4.4	8.5	27.9	19.1	15.1	13.2	10.4	8.0
14	0.61	0.6	1.3	1.1	5.1	4.7	5.1	5.7	4.6	5.0
15	0.37	0.0	0.0	0.8	2.0	2.2	3.4	2.8	2.2	1.9

Table S3. Nicotine plasma concentrations of individual participants before baseline correction after use of a conventional cigarette with their nicotine metabolic ratios (NMR).

Participant	NMR	0 min	1 min	2 min	4 min	6 min	8 min	10 min	12 min	30 min
1	0.43	2.0	1.4	7.5	0.1	1.4	0.1	0.0	0.1	0.1
2	0.17	8.5	3.5	7.3	0.7	10.6	8.2	1.7	1.0	3.0
3	0.16	17.9	19.1	10.0	3.6	30.1	31.4	4.1	1.9	9.3
4	0.44	26.7	20.6	16.4	11.1	55.0	47.6	10.4	13.1	34.3
5	0.61	30.3	21.3	21.6	19.0	56.7	33.3	10.2	11.7	46.2
6	0.67	24.4	20.3	21.2	18.3	34.1	21.9	6.6	7.2	35.5
7	0.52	24.8	18.1	19.5	15.6	26.1	13.6	5.3	6.1	27.2
8	0.67	23.0	14.7	18.6	16.7	20.8	12.7	4.6	5.9	24.3
9	0.26	13.4	13.1	16.1	10.9	15.1	7.3	2.3	4.9	12.0
10	0.70	2.0	1.4	7.5	0.1	1.4	0.1	0.0	0.1	0.1
11	0.33	8.5	3.5	7.3	0.7	10.6	8.2	1.7	1.0	3.0
12	0.46	17.9	19.1	10.0	3.6	30.1	31.4	4.1	1.9	9.3
13	0.51	26.7	20.6	16.4	11.1	55.0	47.6	10.4	13.1	34.3
14	0.61	30.3	21.3	21.6	19.0	56.7	33.3	10.2	11.7	46.2
15	0.37	24.4	20.3	21.2	18.3	34.1	21.9	6.6	7.2	35.5

3. Craving

Acute craving at the different time points are listed in Tables S4 (for HTP 1), S5 (for HTP 2), and S6 (for conventional cigarette).

Table S4. Acute craving for HTP 1 (single question answered on a scale from 1 to 7) over the study period per participant.

Participant	1 min	2 min	4 min	6 min	8 min	10 min	12 min	30 min
1	4	5	5	2	3	1	1	1
2	3	5	5	4	2	1	3	5
3	7	7	7	0	2	1	3	6
4	6	5	4	3	3	3	4	4
5	7	5	5	1	1	1	1	4
6	-	5	3	1	1	2	3	3
7	4	4	3	3	4	4	4	4
8	6	6	5	2	1	1	1	1
9	5	4	4	2	3	4	4	6
10	6	6	5	2	2	3	3	3
11	4	4	3	2	2	2	2	2
12	2	2	2	1	1	1	1	2
13	5	5	4	1	1	1	3	2
14	5	5	3	1	1	1	1	2
15	3	4	4	1	1	1	1	1

Table S5. Acute craving for HTP 2 (single question answered on a scale from 1 to 7) over the study period per participant.

Participant	1 min	2 min	4 min	6 min	8 min	10 min	12 min	30 min
1	4	4	0	1	1	1	1	1
2	3	4	6	2	3	2	1	4
3	5	5	2	1	1	2	2	5
4	6	5	4	4	5	5	4	5
5	2	2	2	1	1	1	1	1
6	3	3	2	1	1	1	1	1
7	4	4	3	3	3	3	3	3
8	2	2	1	1	1	1	1	1
9	5	4	5	4	5	5	5	6
10	5	2	1	1	2	1	1	1
11	5	4	2	1	1	1	1	1
12	1	1	1	1	1	1	1	1
13	6	5	3	1	1	1	1	2
14	1	1	1	1	1	1	1	1
15	1	1	1	1	1	1	1	1

Table S6. Acute craving for cigarettes (single question answered on a scale from 1 to 7) over the study period per participant.

Participant	1 min	2 min	4 min	6 min	8 min	10 min	12 min	30 min
1	1	1	1	1	1	1	1	1
2	6	4	3	1	1	1	1	2
3	7	7	2	1	1	2	3	4
4	5	5	3	1	1	1	1	1
5	4	1	1	1	1	1	1	1
6	3	1	1	1	1	1	1	1
7	3	3	4	3	3	2	2	2
8	5	2	1	1	1	1	1	1
9	3	3	3	2	2	2	2	3
10	3	2	1	1	1	1	1	2
11	2	2	1	1	1	1	1	1
12	2	2	1	1	1	1	1	2
13	2	2	2	1	1	1	1	1
14	3	3	1	1	1	1	1	1
15	5	4	2	1	1	1	1	1

4. Side effects

Side effects were recorded at baseline, at 5 min and at 30 min as shown in Figure S2. At baseline and at 30 min, reported side effects were low.

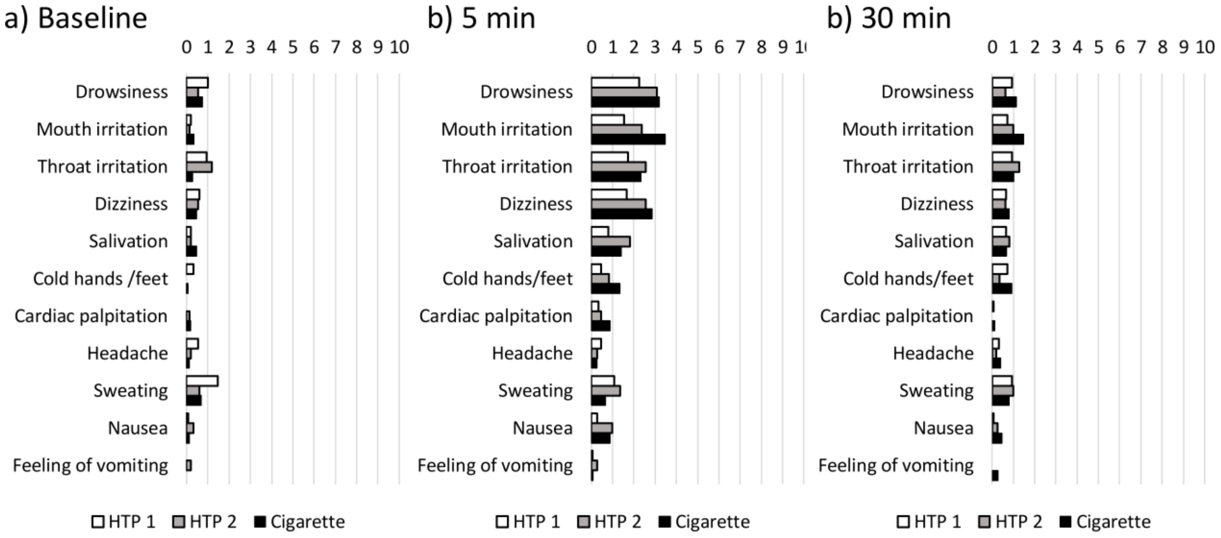

Figure S2. Side effects reported at a) baseline, b) 5 min or c) 30 min on a visual analog scale (VAS) ranging from 0 (no effect) to 10 (strong effect).
